# Spinal Gout Diagnosed by Dual-Energy CT: A Case Report of Prior Misdiagnosis

**DOI:** 10.7759/cureus.90428

**Published:** 2025-08-18

**Authors:** Yao Tang, Jing Fu, Peng Zhu, Jingzhe Ding, Juncai Deng

**Affiliations:** 1 Radiology, Chengdu Second People's Hospital, Chengdu, CHN; 2 Nursing, Jingdong Medical Area of PLA General Hospital, Beijing, CHN; 3 Spinal Surgery, Chengdu Pidu District Hospital of Traditional Chinese Medicine, Chengdu, CHN; 4 Orthopedics, Chengdu University of Traditional Chinese Medicine, Chengdu, CHN

**Keywords:** a case report, differential diagnosis, dual-energy ct, spinal gout, misdiagnosis

## Abstract

It is commonly underestimated how widespread spinal gout can be; in the early phases, it might manifest without symptoms, or the pain it causes could be mistakenly assigned to other conditions that are more commonly seen. The case study outlined in this article revolves around a 35-year-old woman who endured persistent lower back pain over a two-year period, through which she pursued care from multiple medical facilities and specialists, yet saw little to no relief. Lately, her health has worsened, leading to the emergence of widespread pain in both of her legs. A diagnosis of spinal gout was established through dual-energy CT, and the patient was subsequently treated with Febuxostat to reduce uric acid levels, leading to a resolution of her pain. After monitoring for 15 months, symptoms did not reappear. The utility of dual-energy CT in diagnosing and managing spinal gout is highlighted in this paper, as evidenced by the case presented. Furthermore, it is crucial to recognize that the clinical manifestations of spinal gout are often atypical, warranting heightened awareness and consideration in clinical practice.

## Introduction

Spinal gout was first documented by Kersley et al. in 1950 [1]. Currently, there is a scarcity of large-scale epidemiological data regarding its incidence; the existing literature predominantly comprises case reports. Diagnosis is primarily achieved through surgical intervention, although some cases are identified based on clinical presentation, imaging studies, biopsy, or aspiration, with very few diagnosed via dual-energy CT (DECT) [2]. DECT employs two levels of radiation energy to image soft and hard tissue structures, enabling the identification of specific materials and molecules [3]. DECT can detect peripheral gouty tophi with a sensitivity of 84.7-91.9% and a specificity of 85.4-93.7% [4,5]. Conventionally, gout has been recognized as primarily affecting the extremity joints, while spinal involvement has been considered an uncommon manifestation. However, recent evidence suggests that spinal gout may be more common than previously thought [6]. Because the clinical symptoms of spinal gout are typically nonspecific [7], they are often misattributed to other, more common disorders. This report presents a case of a patient with spinal gout who was initially misdiagnosed. The patient was first diagnosed with back muscle strain, myofascial pain syndrome, and disc herniation; however, the symptoms did not improve. Ultimately, DECT examination confirmed the diagnosis of spinal gout. Following treatment aimed at reducing uric acid levels, the patient's pain was significantly alleviated.

## Case presentation

A 35-year-old woman presented with a two-year history of persistent lower back pain. During this period, she sought medical attention at various hospitals and departments, underwent rheumatological assessments and imaging studies of the thoracolumbar spine, all of which yielded normal results. She was diagnosed with lower back muscle strain and myofascial pain syndrome, and underwent treatment involving oral NSAIDs, traditional Chinese medicine, and physiotherapy; however, these measures failed to significantly alleviate her symptoms. Approximately two weeks prior to presentation, the patient experienced worsening back pain, with the discomfort extending to both legs; this escalation persisted despite rest. Subsequently, she was diagnosed with a "herniated lumbar disc" at a nearby medical facility. When initial non-invasive therapies failed to yield results, she was referred to our clinic for further assessment and treatment.

**Figure 1:**
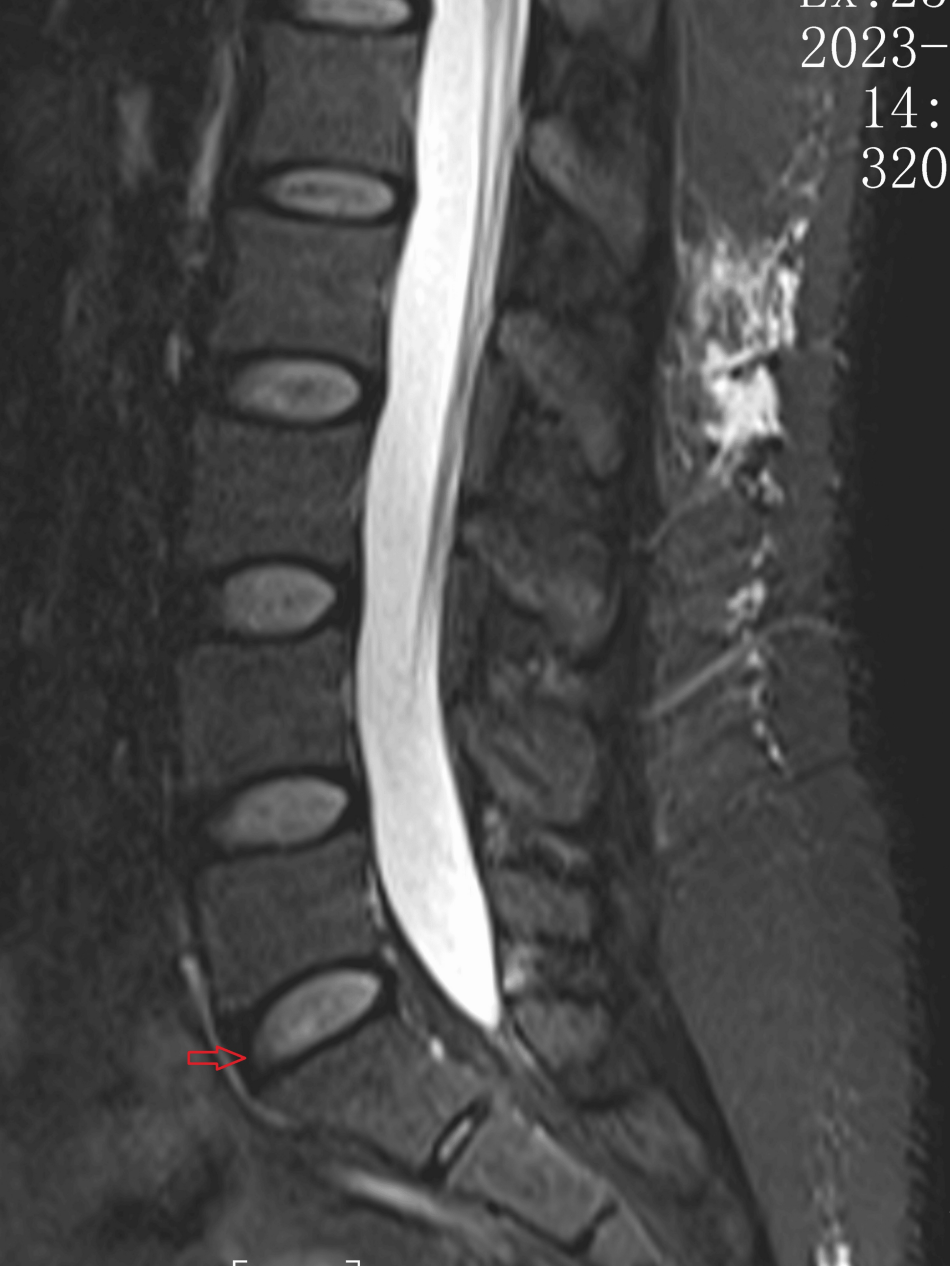
MRI of the lumbar spine The red arrow points to the lumbar intervertebral disc. T2W-FS shows no disc herniation, with posterior fascial edema.

**Figure 2:**
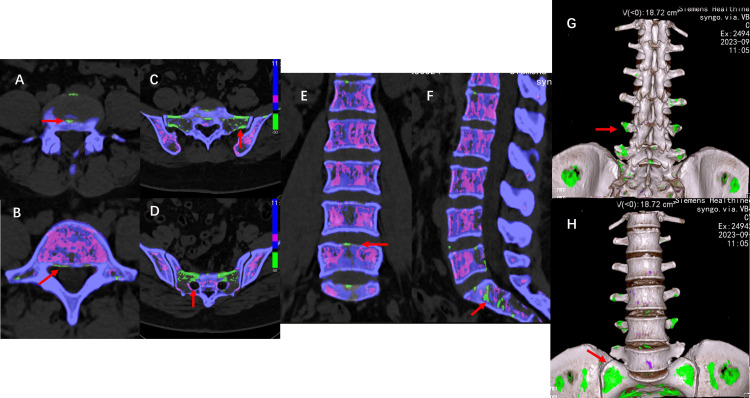
Dual-energy CT at presentation (A) There are deposits in the L4-L5 intervertebral disc; (B) There are deposits on the posterior edge of the L5 vertebra and on the bilateral transverse processes; (C) There are deposits on the S1 vertebra and the bilateral sacral wings; (D) There are deposits surrounding the bilateral sacral foramina; (E) Coronal images reveal deposits along the margins of the vertebra; (F) Sagittal images reveal deposits along the edges of the lumbar and sacral vertebrae, as well as within the sacral vertebrae; (G) 3D posterior aspect of the lumbar spine; (H) 3D Anterior aspect of lumbar spine. The green area indicated by the red arrow shows the urate deposits, with a volume of 18.72 cm³.

**Figure 3:**
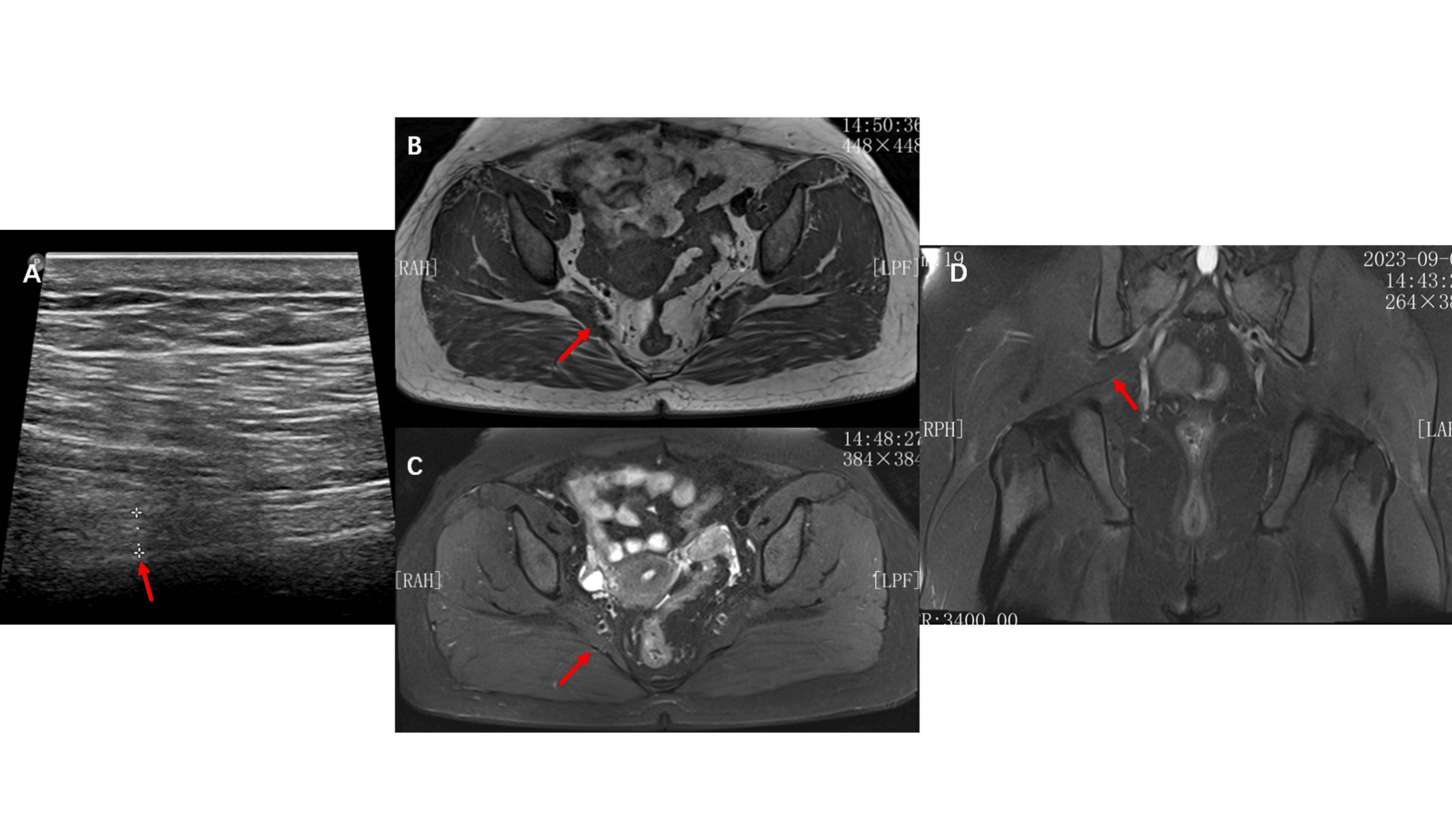
Doppler ultrasound examination of the sciatic nerve and MRI of the buttocks (A) Doppler ultrasound: sciatic nerve (red arrow). (B) MRI T1WI axial, (C) MRI T2W-FS axial, (D) MRI T2W-FS coronal. No abnormalities on MRI. The red arrow indicates the piriformis muscle.

The patient’s medical history included hyperuricemia without acute gout episodes; she had never received uric acid-lowering medications. Physical examination of the lower limbs revealed preserved sensory function and muscular power, with a negative straight leg raise test. Laboratory analysis revealed a blood uric acid level of 464.9 µmol/L. Lumbar spine MRI was unremarkable, showing no evidence of disc herniation or nerve compression (Figure 1). Given her history of hyperuricemia, we hypothesized that her symptoms might be associated with spinal gout. The patient subsequently underwent DECT, which identified small gout deposits within the transverse processes of several lumbar segments, the sacrum, and the iliac bone, with a total volume of approximately 18.72 cm³ (Figure 2A, 2B). At the same time, we performed a color Doppler ultrasound examination of the sciatic nerve and an MRI scan of the buttocks (Figure 3), which ruled out other potential causes of the lower limb pain leading to a diagnosis of spinal gout.

**Figure 4:**
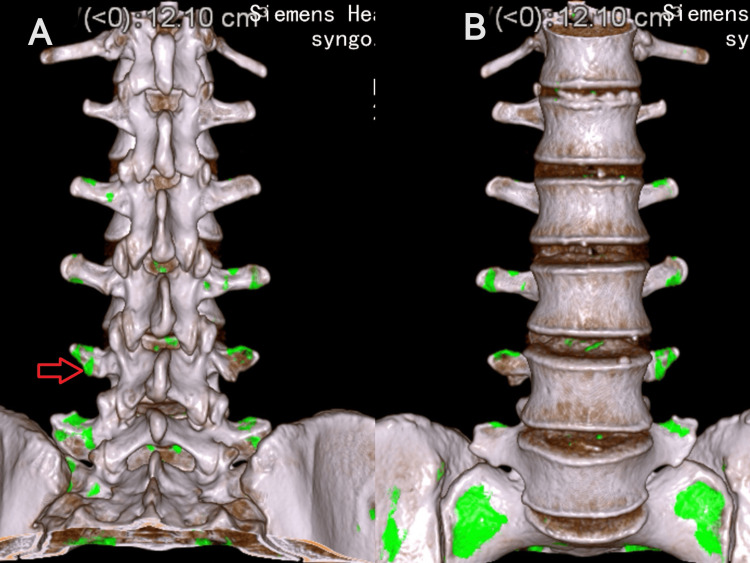
Dual-energy CT for post-treatment evaluation (A) 3D posterior aspect of the lumbar spine; (B) 3D anterior aspect of the lumbar spine. The green area indicated by the red arrow shows the urate deposits, with a volume of approximately 12.10 cm³.

Following the diagnosis, the patient was prescribed oral loxoprofen for pain relief, along with febuxostat to lower uric acid concentrations. Two weeks after initiating treatment, her symptoms had improved significantly, prompting discontinuation of loxoprofen while continuing febuxostat monotherapy. After a 15-month follow-up period, the patient reported complete resolution of her lower back and leg pain. A follow-up DECT showed a reduction in gout deposit volume to approximately 12.10 cm³ (Figure 4A, 4B). The patient's uric acid levels, pain scores, use of analgesics, and use of uric acid-lowering medications before and after the visit are presented in the table below (Table 1).

**Table 1:** Longitudinal monitoring of peri-admission period: dynamics of serum uric acid levels, pain scores, and medication use Serum Uric Acid Level Unit: μmol/L, Reference Range: 150-360 μmol/L. Pain was assessed using the Numerical Rating Scale (NRS). RF=Refused, NA=Not applicable

Stage	Specific time point	Uric acid	Pain score		Uric acid-lowering medications	Special event
			No analgesics were used	Analgesics were used		
23 months before admission	10-18-2021	352.9	3	1	Non-user	Not reported
18 months before admission	03-08-2022	372.60	3	1	Non-user	Not reported
9 months before admission	12-22-2022	330.00	3	1	Non-user	Not reported
At admission	09-04-2023	464.90	5	3	Non-user	Not reported
At discharge	09-23-2023	RF	1	NA	User	Not reported
4 months after discharge	02-02-2024	187.00	1	NA	User	Febuxostat was discontinued thereafter, with a switch to oral Chinese herbal medicine.
8 months after discharge	05-21-2024	407.00	1	NA	Non-user	Oral Chinese herbal medicine
13 months after discharge	06-11-2024	324.00	1	NA	Non-user	Oral Chinese herbal medicine
15 months after discharge	01-09-2025	368.00	0	NA	Non-user	Oral Chinese herbal medicine

## Discussion

First conceptualized in the 1970s, DECT is based on the technique of capturing imagery using two separate energy settings, allowing for the distinction among different materials [8]. Initially, DECT was employed for the diagnosis of gout, specifically to differentiate uric acid kidney stones from other types of renal mineral deposits [9]. Later on, the use of this technique expanded to encompass the identification of monosodium urate (MSU) crystals within joints as well as soft tissue areas [10]. While the conventional benchmark for gout diagnosis has been to locate MSU crystals in synovial fluid [11], contemporary research suggests that this approach is beset with difficulties, prone to a significant number of false negatives, and lacks the ability to extract MSU crystals found in soft tissues. In contrast, DECT has emerged as a highly sensitive and minimally invasive technique for the detection of MSU crystals, offering several advantages, such as the capability to visualize both intra-articular and extra-articular MSU deposits, monitor changes in crystal load over time, and facilitate treatment planning through accurate assessment of treatment responses. The presence of MSU deposition identified through DECT strongly supports a diagnosis of gout, thereby rendering joint aspiration unnecessary [12]. Relative to other diagnostic imaging techniques, DECT shows enhanced precision in detecting gout, with higher accuracy and precision [13]. Furthermore, the 2023 guidelines from the European Alliance of Associations for Rheumatology (EULAR) on imaging for crystal-induced joint diseases clearly suggest that synovial fluid analysis is not invariably an applicable method for diagnosis. In the diagnostic evaluation of gout, DECT is recommended as one of the imaging modalities [14]. Prior studies have established that DECT is more accurate than ultrasound in detecting extra-articular MSU deposition in soft tissues [15].

Gout commonly manifests through the buildup of MSU crystals within joint tissues, as well as in locations outside the joints [16]. The initial manifestation of gout typically occurs as inflammatory arthritis predominantly affecting the lower extremities; however, the involvement of the spinal region is comparatively rare [2]. In 2023, a methodical analysis of records found that since the establishment of the Medline and EMBASE databases up to April 15th of the same year, 315 instances of spinal gout were recorded, with the incidence among males being over four times that among females (4.2:1). It was also highlighted that women affected by the condition were, on average, older than affected men, with their ages averaging at 65 compared to 56 years for men. Surgical procedures were utilized to conclusively determine the medical condition for 150 patients, whereas clinical examination and radiological scans led to the diagnosis in 74 instances, tissue sampling confirmed 34 diagnoses, and needle aspiration identified 13 conditions. A mere 19 patients had their diagnoses confirmed by using DECT, and the methods used to diagnose the rest of the patients were not detailed [2]. The clinical manifestations associated with spinal gout are often non-specific [7], with back pain being the most frequently reported symptom. The diagnostic process can be challenging; however, early identification is essential to mitigate the risk of advancing neurological complications [2]. Although certain studies indicate that spinal gout is more prevalent among adult populations and individuals with elevated serum uric acid levels, other research suggests that age and serum uric acid levels may not consistently serve as reliable predictors of axial gout [17].

Our patient had a history of hyperuricemia but no prior acute gout attacks. He presented with persistent low back pain for two years, having sought treatment across multiple hospitals and specialties without symptom relief. Previous clinicians failed to consider spinal gout as a potential diagnosis. Before admission to our institution, his back pain intensified and progressed to bilateral lower limb radiculopathy refractory to bed rest. Lumbar MRI showed no evidence of disc herniation or neural compression. Given the patient’s hyperuricemia history, we performed DECT, which revealed multiple MSU crystal deposits in the lumbosacral spine. The radicular symptoms were attributed to MSU deposition around the lumbosacral plexus nerves. Thus, this case highlights the critical role of DECT in diagnosing spinal gout. For patients with unexplained back pain unresponsive to conventional therapies - especially those with hyperuricemia - DECT should be considered to investigate spinal gout, establish a diagnosis, and guide targeted treatment.

The diagnostic precision for gout using DECT is notable for its high levels of specificity and sensitivity. Notably, it provides valuable supplementary information for classifying gout [18], even in patients exhibiting negative joint fluid microscopy results. Additionally, DECT appears to hold potential as a predictive instrument, facilitating the creation of bespoke therapeutic approaches matched to the unique load of uric acid in each patient, and acting as a marker for the success of medical treatments [19]. While DECT's sensitivity may be limited in early-stage gout patients, particularly due to a potential threshold concerning the volume or concentration of MSU detectable, it remains a critical diagnostic modality. In cases of spinal gout, where typical clinical manifestations may be absent, it is essential to consider this condition in patients presenting with persistent, unexplained low back pain, particularly those with a history of hyperuricemia and no prior acute gout episodes. Distinctive features of spinal anatomy typically hinder the appearance of external gouty tophi, thereby making DECT a more favorable diagnostic method compared to aspiration or biopsy. Moreover, DECT is instrumental in the continuous monitoring and treatment of gout affecting the spine.

## Conclusions

Spinal gout is a non-specific symptom that often leads to misdiagnosis or a missed diagnosis. Therefore, it is crucial for clinicians to enhance their understanding of spinal gout.

This case highlights the utility of DECT in diagnosing and managing spinal gout. DECT, with its non-invasive approach, high specificity, and quantitative assessment capabilities, not only assists in the diagnosis of spinal gout but also supports its daily management. Nonetheless, we acknowledge certain limitations, including the inability to obtain tissue confirmation and the challenges associated with establishing symptom causality.
